# Host cell state: an overlooked factor impacting the production of influenza A deletion-containing viral genomes and non-infectious particles

**DOI:** 10.1128/mbio.01991-25

**Published:** 2025-09-29

**Authors:** Ilechukwu Agu, Samuel L. Díaz-Muñoz

**Affiliations:** 1 Department of Microbiology and Molecular Genetics, University of California, Davis 8789, Davis, California, USA; 2 Genome Center, University of California, Davis 8789, Davis, California, USA; The Ohio State University, Columbus, Ohio, USA

**Keywords:** virus-virus interactions, flu, non-standard viral genomes, AKT signaling, sociovirology, PI3K, insulin

## Abstract

Influenza A virus remains a global public health threat, prompting the need for novel, broad-spectrum therapeutics. Deletion-containing viral genomes (DelVGs) produced during influenza replication have shown broad-spectrum therapeutic potential via defective interference, where DelVG accumulation depletes the relative abundance of standard viral genomes, diminishing the viral yield needed to sustain pathogenesis. Decades of research have focused on the viral factors affecting the production and maintenance of DelVGs in influenza infections. Surprisingly, the study of host factors that affect the emergence of DelVGs has been neglected. Uncovering host factors that affect DelVG production could help predict infection outcomes based on host state; facilitate the manipulation of host metabolism to increase DelVG production, potentially leading to milder clinical outcomes; and enhance biomanufacturing. The therapeutic potential of increasing *in situ* production of DelVGs is evident, but barriers to progress have persisted for decades, such as a lack of tailored methodologies to reliably quantify defective interference and early research findings that dismissed host cell involvement in the production of DelVGs and the defective interfering particles that carry them. Thus, the molecular mechanism of *de novo* DelVG production remains unknown in spite of evidence implicating host involvement. This review summarizes (i) newly discovered associations between host cell state and influenza DelVGs, (ii) the extensive host-virus metabolic signaling crosstalk that refocused the host as a potential contributor to DelVG/non-infectious particle production, and (iii) the methodological innovations that facilitated these recent discoveries. We conclude by providing an outlook on new avenues in DelVG basic and applied research.

## HOST METABOLISM CAN MODULATE *IN SITU* PRODUCTION OF DELETION-CONTAINING VIRAL GENOMES AND PARTICLES

Within the infected host, influenza A virus has a bipartite existence as intracellular viral genomic segments undergoing replication and assembly into progeny particles and extracellular progeny particles in search of new cells to infect. Owing to the low fidelity of the viral polymerase, there is a striking diversity of mutants among the genomic segments that in turn carry over to the particle level as mutant genomes are packaged into combinatorially variant particles. This within-host pool of closely related yet distinct “individuals” possesses variable fitness upon which natural selection—in the form of host immune responses, antiviral treatments, and virus-virus interactions among the genome segments and particles—acts, favoring the variants better adapted to prevailing conditions (1–8). Fortunately for the sieged host, there is a periodic emergence of nonstandard viral genomes (9); specifically, in the case of influenza, many of these are deletion-containing viral genomes (DelVGs). These DelVGs fail to express the standard, full-length protein, while simultaneously outcompeting standard genomes in their representation within progeny viral particles, either through replication or packaging competition (10–15). Because particles that contain one or more DelVGs do not express the full complement of full-length proteins, they are not capable of sustaining a complete infectious cycle independently and require coinfection by a virion expressing the missing full-length proteins.

DelVGs can also undermine the capacity of standard viral genomes and fully infectious particles to sustain propagation, leading to the term defective interfering particles (DIPs) for those virions that harbor one or more DelVGs. Notably, these truncated segments can encode proteins that can interfere with replication machinery (16). Moreover, DelVGs and DIPs also accelerate the host innate immune response (17), leading to the self-limiting pathogenesis and mild disease severity that characterizes defective interference (18, 19). In fact, host immune modulation by nonstandard viral genomes has been known since the 1970s, in studies that correlated early production of high levels of nonstandard viral genomes with the establishment of persistently infected cells, increased survival, and decreased pathogenicity (20, 21). These initial findings have been corroborated by a body of research detailing how nonstandard viral genomes modulate the host immune system (22–24). The therapeutic potential of on-demand defective interference induction remains unrealized because the mechanism(s) of *de novo* DelVG production during influenza A infection remains unsolved (18, 25–27 ). This mechanism could be elucidated by identifying more factors that influence DelVG production, as groups of these factors likely share common processes involved in DelVG formation. Host cell metabolism exemplifies one such shared process, as evidenced by our studies that recently discovered the impact of metabolic drugs on DelVG production (28, 29).

The host cell’s metabolic signaling state is the most recent of the few known *in situ* modulators of DelVG and DIP production, joining the likes of infection multiplicity (12, 30), virus polymerase gene mutations (19, 31), virus non-structural gene mutations (32, 33), and virus matrix gene mutations (34), as also found for lymphocytic choriomeningitis virus (LCMV) matrix protein (35). In an initial study, we showed that pharmacological disruption of virus-host metabolic crosstalk with an inhibitor of growth metabolic signaling in the host cell modulated *in situ* hallmarks of defective interference early in infection and to varying degrees depending on flu virus strain (28). Pre-exposure of MDCK cells to a PI3Kα inhibitor—alpelisib—prior to A/Texas/50/2012(H3N2) infection significantly increased the relative abundance of non-infectious progeny viral particles at a wide dosage range (2.5, 5, 10, and 20 µM alpelisib), increasing non-infectious particles by ~12% at an early infection stage (18 h post-infection). In contrast, the A/California/07/2009(H1N1pdm) strain did not show changes in the proportion of non-infectious particles but variable changes in the total number of particles produced (28). However, at the genomic level, both strains showed an increasing trend of DelVG production with higher concentrations of alpelisib, with A/California/07/2009(H1N1pdm) infections pre-treated with 20 µM of alpelisib increasing polymerase complex segment DelVGs from 3.11%–4.40% (controls) to 24.67%–40.48%. These findings provided proof of concept that altering the metabolic state of the cell can change non-infectious particle production and DelVG production.

Armed with this proof of concept, we then set out to find other novel metabolic signaling drugs that act through the host to modulate *in situ* hallmarks of DI early in infection by examining the relative production of DelVGs specifically, e.g., controlling for impacts on full-length replication (29). We found that adenosine was a potent and consistent amplifier of DelVG production. Adenosine increased DelVG production in all three polymerase complex segments by 35.00%–80.61% in both tested H1N1 and H3N2 strains and also increased DelVGs in other segments (29). Insulin showed strain-specific effects on polymerase complex DelVGs, increasing DelVG production in the H3N2 subtype (29). Tricarboxylic acid (TCA) cycle inhibitors 4-OI and UK5099 significantly boosted total viral genome production across multiple segments (29), mimicking the Warburg effect observed in tumor cells, where cells forgo oxidation of nutrients to favor increased biomass. These metabolic signaling molecules collectively link host metabolism to DelVG production through their shared impact on altering metabolic signaling pathways within the host cell. In addition to these drugs, a low dose of the mutagenic nucleoside analog precursor Favipiravir increased total viral genome production across H1N1 and H3N2 subtypes, while slightly reducing DelVG proportions in the H3N2 subtype, indicating that nucleotide misincorporation may not be essential for DelVG production (29). Lastly, cyanobacterial extracts selectively and almost completely shut down the production of antigenic segments (hemagglutinin and neuraminidase) in the H3N2 subtype, highlighting the potential of natural products in modulating segment-specific forces that act during replication and capsid assembly (29, 36).

Of the few known modulators of DelVG and DIP production, the host cell metabolic signaling state is unique for being the most therapeutically actionable, i.e., it can be monitored readily and provides targets that are potentially easily druggable. This discovery was made possible in part by the extensively characterized crosstalk between host metabolism and influenza infection outcomes.

## HOST-INFLUENZA INTERACTIONS: A HISTORY OF METABOLIC CROSSTALK

At the cellular level, influenza A induces various metabolic changes within infected cells that favor productive pathogenesis. In a particularly striking example, A/Puerto Rico/8/34(H1N1) infection disrupted proteasomal degradation of hypoxia-inducible-factor-1α (HIF-1α) in the mitochondria of human lung cells (A549) and mouse lung tissue, allowing for accumulation and translocation of this transcription factor (HIF-1α) to the nucleus where it facilitates expression of pro-glycolytic enzymes (37). This aberrant reprogramming of host cell glucose catabolism disrupts the normoxic oxidation of pyruvate by upregulating hexokinase (HK2), pyruvate kinase M2 (PKM2), and pyruvate dehydrogenase kinase (PDK3) (38). This state, similar to that of tumor cells, is characterized by enhanced glycolysis and the redirection of pyruvate from complete breakdown into CO_2_ gas, preserving the reduced-carbon biomass needed to feed anabolic pathways that drive the proliferation of tumor cells (39, 40) or viruses.

Influenza A virus also upregulates host biosynthetic pathways that support viral proliferation via direct binding of the viral NS1 effector protein to the regulatory subunit (p85β) of host class 1a phosphoinositide-3-kinase (PI3Kα) (41). This interaction releases the catalytic p110α subunit to initiate the PI3Kα signaling cascade (42–47), with strain-dependent intensity (28, 44), even in the absence of typical growth factors like insulin. Consequently, NS1’s actions shift host metabolism toward a state marked by increased pools of precursor metabolites (48–50) vital for uninterrupted replication of virion components.

Conversely, host metabolism can equally influence the course of influenza infection. In a straightforward example at the cellular level, growth media supplementation with the Tricarboxylic Acid Cycle inhibitor malonate drove dose-dependent decreases in the total particle yield of viral progeny (51). At the organism level, other metabolic factors and host states such as obesity, diabetes, nutritional status, and pregnancy have been shown to impact susceptibility to influenza infection and follow-on disease severity. For instance, extreme nutritional states of either diet-induced obesity or caloric restriction impaired immune function and increased the risk of complications from influenza infection in mice (52). Additionally, obese patient groups shed higher viral loads, which also contained more virulent mutants relative to the non-obese cohorts in human infections (53, 54). Pregnancy has long been known to be a state associated with more severe infection. More recent research has shown that this is due to the host’s changed immune landscape, which impairs host defenses and facilitates the emergence of more pathogenic viral variants (55, 56).

There is a steadily expanding body of work on how DelVGs affect the host, including the molecular mechanisms underlying host cell detection of the Z-conformation RNA of DelVGs (17); DelVG-induced variation of the host transcriptional program (47); and interferon-independent protection of co-infected influenza defective interfering virus on types I and III interferon-deficient mice (57). Strangely, the reverse is not the case, as evidenced by a striking absence of research into the effects of host cells on DelVG production. In truth, host cell involvement in DI virus production was wrongly dismissed well over half a century ago, and the extensive host-influenza metabolic crosstalk characterized in the intervening decades served to refocus the host as a key contributing factor.

## THE “NOT” STAR: EARLY INQUIRIES WRONGLY DISMISSED HOST CELL INVOLVEMENT IN INFLUENZA A DI VIRUS PRODUCTION

Shortly after the discovery of non-infectious influenza A particles and their antiviral potential (11, 12), scientists speculated about the role of host cells in their production—no doubt in response to mounting evidence of viral sensitivity to perturbation in host cell functions like the TCA cycle (51), glycolysis (58), and vitamin A metabolic signaling (59). However, initial attempts to establish a connection between host parameters and DIP production found only a meandering correlation with virally induced cell damage (60). These early probes also had some limitations, like overlooking the effect of multiplicity of infection (MOI) (61, 62) and the misattribution of control variables (61, 63). As such, these studies were unsuccessful in disentangling host effects from MOI and consistently found MOI to be the primary determinant of DIP production, while the host had no effect. These findings and subsequent support for MOI as the main determinant of DIP production (12, 30) appear to have diverted the collective pursuit for inducers of DIP production away from the host cell. This diversion is evidenced by the abrupt drop-off in research, which failed to resurge despite the emergence of supporting evidence over the subsequent decades.

The intervening decades following the dismissal of host cell involvement in DIP production saw several missed opportunities to refocus the host cell as a potential controlling factor. A recent review (27) observed that the per-segment DelVG profile of flu strains may differ with the cell type infected, citing the disparate DelVG outcomes of A/Puerto Rico/8/34 infection in two independent studies that used different cell types, embryonated eggs (64) and MDCK cells (65). In another case, researchers discovered that the fatty acid and phospholipid profile of the A/Puerto Rico/8/34 envelope differed significantly between infectious and non-infectious particles (66, 67). This discrepancy suggests that lipid-driven changes in host cell membrane rigidity might differentially affect the efficiency with which nascent DelVG and standard genomes are packed into progeny particles. Lastly, influenza A polymerase replication fidelity suffers under low concentration of its ribonucleotide triphosphate (rNTP) substrate *in vitro* (68). Given that internal deletions of DelVGs have the appearance of a replication error product, their *de novo* production may also be driven by low rNTP concentration or other environmental determinants of polymerase physiochemistry and fidelity, such as pH, temperature, rNTP pool balance, choice of metal ion cofactor (Mg^2+^ or Mn^2+^), crowding, and other factors (68, 69) that are regulated by host signaling networks. Moreover, altering the physiochemistry of influenza polymerase through sequence mutations directly affected DelVG accumulation (19, 31), indicating the potential for physiochemical changes induced by various factors, including those originating from the host, to yield similar effects.

The above-mentioned examples are just a glimpse into a broader pool of uncurated findings implicating host involvement in DIP production, which has been slow to rekindle interest in this once-dismissed area (60–63). A persistent barrier to discovering more factors shaping DIP production is the absence of methodologies capable of not only quantifying DI phenomena at sufficient resolution but also doing so with precision and high throughput. In fact, assays enabling precise quantification of DelVGs (28, 70, 71) and non-infectious viral particles (28, 72, 73) have only recently been developed. We now turn to review past and current methods of quantifying DelVGs and DIPs, with an emphasis on methodological shortfalls and recent innovations that promise to revitalize research in this field.

## METHODOLOGICAL APPROACHES TO QUANTIFYING DEFECTIVE INTERFERING PHENOMENA, PAST AND PRESENT

This review focuses on DelVGs and DIPs. From a methodological perspective, we focus on non-infectious particles as opposed to DIPs. Non-infectious particles, which cannot complete a full cycle of replication independently, can lose their infectious ability for a number of reasons, including DelVGs, gene-lethal mutations, or failure to express a segment (semi-infectious particles *sensu* [73]), among others (reviewed in reference 74). Fundamentally, there are no methods to distinguish the different potential types of non-infectious particles at the virion level; thus, we use the term non-infectious particle to precisely convey what the methods measure. Standalone titration of fully infectious particles (FIP) or non-infectious particles (NIP) provides an incomplete representation of disease state due to their entangled antagonism, which shapes influenza A pathogenesis. The same rings true at the genome level, where standalone counts of SVGs or DelVGs overlook the entangled effects of both segment types on disease progression and outcomes. This is why interference is best quantified in terms of the ratios or proportions of different viral sub-groups relative to each other—both at the particle and genome level. Options to quantify interference at the particle level include NIP:FIP ratio or NIP relative abundance. In the same vein, genome-level interference can be represented via the DelVG:SVG ratio or DelVG relative abundance.

### Particle-level DI: quantifying non-infectious particles

Particle-level interference during influenza A virus infection was first quantified as the ratio of FIPs to total particles (60). FIPs were titrated via plaque assay (75) and reported as the number of plaque forming units (PFU), while total particles were titrated via hemagglutination assay (76) and reported as the number of hemagglutination units (HAU). However, the PFU:HAU ratio had low precision because HAU is only an approximation of total particles, not an actual count. Additionally, the PFU:HAU ratio did not directly report on interference but rather productive infectivity, meaning that observed changes in the metric may or may not be due to interference.

The infectious center reduction assay (77) was the breakthrough assay that first quantified an interference metric from influenza A virus infection, albeit indirectly and with imprecision. Co-infecting a viral sample (sample A) of known PFU with a viral sample (sample B) of unknown titer and then measuring the reduction in titer of sample A allowed researchers to quantify sample B’s interference as defective interfering units (DIU/mL) (62, 77, 78). The DIU:PFU ratio could now be derived to report on interference in an influenza A infection. However, the DIU metric is imprecise because it does not directly measure interfering particles but derives interference from another metric. A similar assay, the plaque reduction assay (79), has been used more recently to quantify the activity of influenza DIPs. An alternate approach developed for respiratory syncytial virus (RSV) was the colorimetric assay that stained cells that were protected by DIPs (80).

We developed the cluster-forming assay to titrate influenza A viral particles (28). This assay builds on the method pioneered by Brooke et al. (73), which uses single-cell immunofluorescence to reproducibly titrate non-infectious and infectious influenza A particles directly, in a physiologically relevant adherent cell monolayer model. The methodological innovation of the cluster-forming assay lies in (i) the replacement of the plaque assay’s solid agar overlay with a semi-solid overlay that is aspirated post-assay, which facilitates high-throughput immunofluorescence staining and imaging of the monolayer; and (ii) the automated computational image analysis pipeline. In the resulting immunofluorescence image, infectious and non-infectious particles are clearly resolvable based on whether an infection event has propagated to adjacent cells (fully infectious) or remains confined to a single cell (non-infectious) (28). Fully infectious and non-infectious particles are then summed to yield the total particles, which is used to divide the number of non-infectious particles to derive the relative abundance of non-infectious particles; a precise metric of defective interference based on the actual count of infective and non-infective particles (28).

### Genome-level DI: quantifying deletion-containing viral genomes

Progress in particle-level defective interference quantitation initially outpaced DelVG quantitation, with the infectivity-hemagglutination ratio (PFU:HAU) (60) entering use a full 20+ years ahead of the discovery of influenza A DelVGs. Initially termed “subgenomic RNAs,” DelVGs were discovered via PAGE of phenol-extracted viral RNA (77, 81). They were quantified either qualitatively by the presence or absence of a gel band (81) or quantitatively by determining the molar ratios of standard and DelVG segments relative to a reference segment (77). This quantitative method involved creating an autoradiograph from a PAGE gel of radioactively labeled RNA segments, where band intensities on the autoradiograph correlated with the amount of RNA and were analyzed using densitometry to measure the counts per minute (CPM) for each band. These CPM values were then compared to the CPM of a reference segment to determine the relative abundance of each RNA segment (77). Although pioneering, the molar ratio method was imprecise because CPM is only an approximation of total genomes per segment, not an actual count.

Quantitative PCR (qPCR) allows for influenza A virus DelVG detection via the use of internally binding primer sets that target regions flanking the known deletion sites in the viral genome (82). This allows the amplification of both full-length and deletion-containing genomes, but detection in this manner is limited to DelVGs of known deletion sites. As such, a forward approach to detect all possible deletion junctions in any given viral sample will require a vast amount of custom primer sets, which will significantly reduce throughput and prove technically difficult. Moreover, the specific DelVGs that qPCR manages to detect are subject to imprecise quantitation due to (i) PCR amplification bias and (ii) the inference of genomic cDNA production from the probe fluorescence instead of being directly counted.

More recent workflows to detect internal deletions in influenza A genomic segments pair next- or third-generation, long-read sequencing with downstream bioinformatics. Long-read sequencing in particular allows investigators to classify sequenced reads on the basis of internal deletions or other recombination events they harbor, but these recombination events must first be flagged via the alignment of sequenced reads to the matching reference influenza A genome. DelVG-tailored sequence alignment tools like ViReMa (83–85), DI-tector (86), and VODKA2 (87), as well as other aligners like TopHat2 (88), STAR Aligner (89), and HMMER (90) have been successfully used to detect deletion-containing reads in short- and long-read sequencing data sets (15, 16, 19, 28, 70, 71, 91). However, both short- and long-read sequencing platforms currently boast different efficiencies and capabilities with regard to detecting and quantifying influenza A DelVGs.

The highest throughput and accuracy (nearly 100.0% per base) for influenza A virus sequencing is achieved with the next-generation sequencing (NGS) short-read sequencing Illumina platform (92, 93). However, the need for sequencing library fragmentation in NGS makes it impossible to distinguish fragmented DelVG and full-length segments. Consequently, DelVG deletion junction mapping is the current limit of the Roche/454 (15) and Illumina (94) NGS platforms regarding DelVG identification. Experimental and computational artifacts from physical DNA fragmentation can be mitigated by various methods, such as using simulated control data sets to validate deletion breakpoints (94) or employing ClickSeq to avoid physical fragmentation and enzyme-mediated ligation of sequencing adapters. In ClickSeq, sequencing library preparation starts with a reverse transcriptase reaction using semi-random DNA primers, deoxyribonucleotides, and a 3′-modified nucleotide analog that randomly terminates DNA synthesis, producing variably sized 3′-blocked cDNA fragments similar to dideoxy-Sanger sequencing (95). These fragments are then purified and reacted with sequencing adapters bearing a 5′-modified chemical group, which binds both molecules at their 3′ and 5′ ends into ssDNA substrate for PCR amplification to generate a viral cDNA library (95). Despite ClickSeq’s advantages, the dependence on library fragmentation—whether physical or non-physical—limits NGS platforms to reporting the location and abundance of deletion breakpoints per segment (70). Fortunately, advancements in long-read sequencing platforms have allowed researchers to overcome the limitations of short-read sequencing data.

End-to-end sequencing of the influenza A virus genomic segments on the Oxford Nanopore Technologies long-read sequencing platform has made it possible to detect the deletions of all possible lengths in any given viral segment and, therefore, classify and count the number of standard and deletion-containing genomes in a given sample (28)—a feat as yet not achieved with short read genomic data of influenza A. Additionally, the current generation Oxford Nanopore hardware and software have a modal per-base accuracy of 97.21% for influenza sequencing, a 1.35% point improvement from the previous generation (96). This trend of improvement puts Oxford Nanopore on track to rival Illumina in per-base accuracy in the coming years and outperform Illumina if the short-read platform is unable to expand its capabilities to include long-read sequencing.

In recent influenza A DelVG investigations, cDNA synthesis followed by PCR amplification with universal influenza primers has become the preferred method of sequencing library preparation (15, 16, 19, 28, 71). However, PCR amplification introduces a risk of bias (97, 98), affecting the accuracy of segment and deletion junction counts. To enhance precision, researchers now use DNA primers with unique molecular identifiers (UMIs) during the reverse transcriptase reaction, incorporating UMIs into viral cDNA before amplification. In the bioinformatics process, amplicons with identical UMIs are collapsed into a single representative read, accurately reflecting the true count of viral cDNA, and can also be used to increase the per-base accuracy through consensus of multiple reads sharing the same UMI (99). Finally, advancements in direct RNA sequencing on third-generation sequencing platforms (100) promise further innovation by eliminating the need for PCR and UMI deduplication by quantifying native RNA molecules (16). In lieu of the mainstream adoption of direct RNA sequencing in the Influenza A DelVG research, which is hampered by a lack of officially supported multiplexing (101), UMI-deduplicated amplicons are classified as DelVGs and SVGs and divided to obtain a precise relative abundance of DelVGs and an accurate count of mapped deletion junctions based on the actual count of genomic segments (28).

## CONCLUSION AND OUTLOOK

The mechanism(s) of *de novo* DelVG production during influenza A infection remains unsolved. The discovery of related factors that impact the production of DelVGs and non-infectious viral particles holds the promise of informing the chain of events that lead to the *de novo* emergence of DelVGs. The potential for the host cell to modulate influenza A DelVG production is a logical target of inquiry because of the strong dependence of viral pathogenesis on cell machinery. Host effects were pursued for a time (60–63) but abandoned in the wake of findings that increasingly pointed to the multiplicity of DIPs in the inoculum as the main contributor of DelVG production (30, 62, 63). There has also been an absence of a concerted effort to uncover the causes and mechanisms behind DelVG *de novo* emergence in the subfield of influenza A DelVG research—likely due to the absence of methodologies capable of quantifying DelVGs and non-infectious particles with the requisite precision, at meaningful resolutions, and with sufficient throughput. Fortunately, methodological innovations—such as the cluster-forming assay to titer non-infectious particles (28, 72) and the combination of long-read genome sequencing with unique molecular identifier deduplication to titer DelVGs directly (28, 70)—have led to the discovery of associations between the host cell state, particularly metabolism and metabolic signaling, and DelVG production.

The renewed evidence that host cell metabolism can affect DelVG production opens up several avenues for basic and applied research. There is a new class of mechanisms within the host to investigate DelVG production, which should assist in the search to elucidate how exactly DelVGs form in the host cell in the first place. Furthermore, these findings can inform the dynamics of virus-virus interactions, as the host context can alter the composition of the DelVG population (2, 7), which can alter the course of the infection and has potential implications for viral host jumps. Host involvement in DelVG production also presents many possibilities for diagnostics, therapeutics, and their manufacturing. An exciting possibility is to link host cell states that are particularly susceptible to severe influenza infection with existing metabolite screens as a diagnostic of infection severity to prioritize medical resources. The induction of host cell states that steer flu pathogenesis toward milder outcomes using metabolic drugs is a novel therapeutic approach that could take advantage of existing, approved drugs. However, this approach comes with an important caveat: timing of nonstandard viral genome production is crucial, and late administration can have neutral effects or even exacerbate disease (22). Thus, more research is needed to determine the feasibility of inducing host states that can alter pathogenesis. Finally, as therapeutic interfering particles are identified and manufactured, compounds that can boost the cell production of DelVGs could be a very useful tool to scale production of DelVG-based therapeutics. Hopefully, these new methodologies and discoveries will multiply into breakthroughs that finally allow mapping of mechanisms that underlie *de novo* DelVG production and harnessing this information to stem the ongoing burden of influenza epidemics and pandemics.
